# Polysaccharide Composite Alginate–Pectin Hydrogels as a Basis for Developing Wound Healing Materials

**DOI:** 10.3390/polym16020287

**Published:** 2024-01-20

**Authors:** Galina A. Davydova, Leonid L. Chaikov, Nikolay N. Melnik, Radmir V. Gainutdinov, Irina I. Selezneva, Elena V. Perevedentseva, Muhriddin T. Mahamadiev, Vadim A. Proskurin, Daniel S. Yakovsky, Aurel George Mohan, Julietta V. Rau

**Affiliations:** 1 Federal State Institution of Science Institute of Theoretical and Experimental Biophysics of the Russian Academy of Sciences (ITEB RAS), Institutskaya St., 3, Pushchino 142290, Moscow Region, Russia; davidova_g@mail.ru (G.A.D.); selezneva_i@mail.ru (I.I.S.); 2 Federal State Budgetary Institution of Science P.N. Lebedev Physical Institute, Russian Academy of Sciences, Leninsky Prospekt, 53, GSP-1, Moscow 119991, Russia; chaykovll@lebedev.ru (L.L.C.); melniknn@lebedev.ru (N.N.M.); perevedencevaev@lebedev.ru (E.V.P.); muhriddin.mahamadiyev@gmail.com (M.T.M.); 3 Federal Research Centre “Crystallography and Photonics” of the Russian Academy of Sciences, Leninsky Prospekt, 59, Moscow 119333, Russia; radmir@crys.ras.ru; 4 Pushchino Branch of Federal State Budgetary Educational Institution of Higher Education “Russian Biotechnology University (ROSBIOTECH)”, Nauki Ave. 3, Pushchino 142290, Moscow Region, Russia; vadimapro@bk.ru; 5 Department of Biotechnology, Institute of Natural Science, Federal State Budgetary Educational Institution of Higher Education “Tula State University”, Lenin Ave. 92, 9th Academic Building, Tula 300012, Russia; yackowsckay@mail.ru; 6 Faculty of Medicine and Pharmacy, University of Oradea, 10 P-ta 1 December Street, 410073 Oradea, Romania; mohanaurel@yahoo.com; 7 Department of Neurosurgery, Clinical Emergency Hospital Oradea, 65 Gheorghe Doja Street, 410169 Oradea, Romania; 8 Istituto di Struttura della Materia, Consiglio Nazionale delle Ricerche (ISM-CNR), Via del Fosso del Cavaliere 100, 00133 Rome, Italy; 9 Department of Analytical, Physical and Colloid Chemistry, Institute of Pharmacy, I.M. Sechenov First Moscow State Medical University, Trubetskaya 8, Build. 2, Moscow 119048, Russia

**Keywords:** wound healing, gels, hydrogels, alginate, pectin, polysaccharides, damaged tissues

## Abstract

This article presents materials that highlight the bioengineering potential of polymeric systems of natural origin based on biodegradable polysaccharides, with applications in creating modern products for localized wound healing. Exploring the unique biological and physicochemical properties of polysaccharides offers a promising avenue for the atraumatic, controlled restoration of damaged tissues in extensive wounds. The study focused on alginate, pectin, and a hydrogel composed of their mixture in a 1:1 ratio. Atomic force microscopy data revealed that the two-component gel exhibits greater cohesion and is characterized by the presence of filament-like elements. The dynamic light scattering method indicated that this structural change results in a reduction in the damping of acoustic modes in the gel mixture compared to the component gels. Raman spectroscopy research on these gels revealed the emergence of new bonds between the components’ molecules, contributing to the observed effects. The biocompatibility of the gels was evaluated using dental pulp stem cells, demonstrating that all the gels exhibit biocompatibility.

## 1. Introduction

The processes within wound healing represent a complex set of defensive reactions in an organism, the development of which occurs in response to tissue damage. The organism’s defensive reactions manifest in the form of both destructive and regenerative processes in the wound area, along with general reactive changes in the organism. The wound healing process involves the same cellular elements that determine the dynamics of the wound process (inflammation, tissue granulation, and epithelization). In a comprehensive approach to the problem of locally treating wounds and burns, a significant emphasis is placed on treatment using a low dosage of pharmacological substances.

Currently, one of the most promising directions in the development of wound-healing materials is the use of polymeric systems of natural origin based on polysaccharides. Representative members of this class include sulfated glycosaminoglycans from animal connective tissue, namely: hyaluronic acid, sulfated galactans from red algae, alginic acid and its salts, pectins, and some hemicelluloses of plants [1]. These biopolymers, being active biocompatible systems, are capable of the prolonged controlled stimulation of reparative tissue regeneration [2,3,4]. Wound healing is induced by polysaccharides through the stimulation of cellular activity (cell motility, proliferation, and the synthesis of extracellular matrix of cytokines and growth factors). An important advantage of using biopolysaccharides over synthetic analogs and other representatives of the biopolymer class in the creation of wound-healing materials is the relatively low cost and availability of renewable raw material sources [5].

The advancement of modern wound-healing materials involves the coordination and control of the growth of various tissue and cell types, influenced by both systemic and local micro-environments. During the process of wound healing, maintaining moist conditions at the wound site has been found to accelerate the healing rate compared to dry conditions [6]. This enhancement is attributed to improved cell migration, including the movement of fibroblasts and keratinocytes, leading to accelerated re-epithelialization. Additionally, moist conditions prevent dehydration, the breakdown of dead tissue and fibrin, increased proliferation of fibroblasts and keratinocytes, enhanced angiogenesis, and the stimulation of collagen synthesis [7,8]. It has been shown that occlusive dressings that maintain a moist environment contribute to enhanced re-epithelialization and the healing of chronic wounds. Moreover, they help to reduce inflammation, pain, infection, and scarring. Wet alginate gel dressings, such as Algosterile (J&J, Ascot, Berks, UK) and Sorban (Dow, Midland, MI, USA), are utilized to absorb exudate from skin ulcers. Isotonic saline gels, like Normlgel (Scott Health Care, High Wycombe, UK), serve as moist protective dressings to promote the healing process [9].

Certain natural and biocompatible polysaccharides, such as alginate, are currently employed as wound dressings due to their inherent adhesive properties and their capacity to stimulate the healing process [10]. Unlike other polymers, they can adhere to the wound site [11], absorb exudate, and transform their physical state into a hydrogel capable of covering and maintaining adequate moisture in the wound bed [12]. This, in turn, allows efficient oxygen circulation, fostering increased cell and tissue regeneration, while reducing bacterial load [13]. Furthermore, alginate can induce the production of cytokines by human monocytes by interacting with mannuronic residues of alginic acid. This pro-inflammatory stimulus is particularly beneficial in the treatment of chronic wounds when macrophages may not have reached an appropriate state of differentiation. In such cases, the healing process can take advantage of exogenous pro-inflammatory stimuli to which macrophages are susceptible [14]. Hydrogels containing alginate and pectin may serve as important tools for creating an optimal microenvironment to promote wound healing. Such materials not only provide a substrate for wound cells, but also stimulate cellular activation and promote the formation of an extracellular matrix, cytokines, and growth factors. The moist conditions facilitated by alginate dressings contribute to faster healing and help prevent complications. Innovative materials and techniques, like these, have the potential to enhance wound care outcomes by facilitating healing and reducing the risk of complications. Pectin is a complex polysaccharide primarily composed of esterified $D\overset{´}{\alpha }$-galacturonic acid residues in the chain (1–4), interrupted by short rhamnose insertions that disrupt the chain–helix configuration. It also contains other neutral sugars as side chains [15]. A distinctive feature of pectin, compared to other bioactive natural polymers (e.g., gelatin, fibrin), is the absence of endogenous cell-adhesive and cell-proteolytic sites. This absence allows for the precise introduction of specific biochemical moieties into the bioinert main chain of the polymer [16], enabling control over cell fate and the separation of their effects on cell behavior [17]. Due to its chemical nature, pectin exhibits good biocompatibility and availability. Its medical applications are attributed to both its ability to form gels and its synergistic interaction with other polysaccharides, including alginate.

Composite alginate–pectin hydrogels are particularly intriguing due to the interactions between polysaccharides, leading to the formation of complexes that significantly enhance biological activity through synergistic effects on cell receptors and improved rheological properties [18]. The nature of this synergistic interaction between pectin and alginate in composite gels is not fully understood, but it appears to be specific rather than based on incompatibility or exclusion effects. This specificity is attributed to the heterogeneous association of the poly-G-blocks of alginate and the low-charge methyl ester regions of pectin packing together into rigid ribbons [19,20,21]. Circular dichroism studies conducted by the authors of [20] confirmed that the spectral changes associated with mixed-gel formation had the same general shape and peak maxima as those observed for calcium-induced gel formation, resembling egg-crate models. The nearly mirrored ratio between polygalacturonic acid in the pectin chain and poly-G in the alginate chain, coupled with the observation that pectin with a high degree of esterification (DE) and alginate with a high α-L-guluronic acid (G) content resulted in the strongest gels, led the authors [20] to suggest that blocks of methyl-esterified polygalacturonic acid bind to blocks of poly-G. Molecular models presented in [20] also demonstrate that ribbons of poly-G and methyl-esterified polygalacturonic acid can pack together in parallel double-crystalline arrays.

In addition to the inherent nature of alginate and pectin chains, several other factors, including concentration, hydro-colloid ratio, ions present, sugar concentration, and pH, play a crucial role in determining the gel strength. For mixed gels composed of high DE pectin and alginate with either a high or low M/G ratio (where M is β-linked -D-mannuronic acid), the maximum gel strength is observed at a ratio of approximately 1:1 [19,20,21]. As mentioned above, pH is a crucial factor influencing gel characteristics. Gel strength tends to increase as pH decreases from 3.5 to 3.0, after which the gel strength remains relatively constant [20]. Additionally, the melting point of gels decreases with an increase in pH [19].

In contrast to the extensive study of rheological behavior, the microstructure of mixed alginate/pectin gels has received less attention. A recent investigation [22] has focused on the microstructure of pectin gels [22]. The study reported on the microstructure and rheological behavior of pure pectin gels with a high or low DE, as well as their mixed gels. The pure gels were shown to exhibit a similar microstructure regardless of differences in DE and, consequently, in the gelation mechanism. The gels consisted of open meshes with large pores in the range of 500 nm and mesh filaments with similar characteristics. The large pores suggested an aggregated mesh structure at the supramolecular level rather than the molecular level. To determine whether synergism could be achieved for pectin samples with a high or low DE, various gelation mechanisms were activated in the mixed system by altering sucrose and calcium concentrations. The results indicated that variations in environmental complexity led to significant or no synergistic effects.

The rheological behavior of mixed alginate/pectin gels has been investigated in various studies [19,20,21,23,24]. Based on measurements of the characteristic viscosity of pectin and alginate with high M/G ratio [21], it was hypothesized that aggregation is the stage preceding gel formation. However, the parameters of these aggregates and their influence on biological activity have not been thoroughly investigated.

The objective of the current study was to elucidate the mechanism of interaction between alginate and pectin in the gel resulting from their mixture. To achieve this goal, the non-crosslinked gels of both components and their mixture were examined using atomic force microscopy (AFM), dynamic light scattering (DLS), and Raman spectroscopy (RS). Additionally, the biocompatibility of the gels was assessed using dental pulp stem cells (DPSC).

## 2. Materials and Methods

### 2.1. Preparation of Gels

In this study, pectin from citrus peel (P9136-100G, Sigma-Aldrich, Burlington, MA, USA) and alginic acid sodium salt from brown algae, medium viscosity (A2033-250G, Sigma-Aldrich), were used. Solutions of sodium alginate at a concentration of 20 mg/mL, pectin at a concentration of 20 mg/mL, and alginate/pectin mixture at a concentration of 10 + 10 mg/mL were prepared. These solutions were prepared by dispersing the respective powders in distilled water at a temperature of 90 °C and stirring for 60 min using a top-drive stirrer (US-2000A ULAB, St. Petersburg, Russia).

To elucidate the mechanism of action of the alginate/pectin mixture gel, the gels obtained from both components and the mixture were investigated using atomic force microscopy, dynamic light scattering, and Raman light scattering spectroscopy.

### 2.2. Atomic Force Microscopic Imaging

Samples for AFM study were prepared on freshly prepared mica substrates obtained by splitting a mica plate. A thin (~30 μm) layer of 2% alginate, pectin, or their 1:1 mixture of the same concentration was applied to them and dried in vacuum at 10^−2^ mm Hg using a nitrogen trap.

The membrane surface morphology was studied with atomic force microscopy in intermittent-contact mode with an NTEGRA Prima instrument (NT-MDT Spectrum Instruments, Moscow, Russia) using HA_FM silicon cantilevers (Capella LLC, Moscow, Russia). AFM studies of the surface of the samples were performed under controlled conditions in the measuring complex “TRACKPORE ROOM-05”, with a purity class of 5 ISO (100), maintaining humidity at 40 ± 1%, and temperature at 22 ± 0.05 °C.

### 2.3. Dynamic Light Scattering

Since the dynamics of gels lie in the frequency range from a few hertz to kilohertz, the dynamic light scattering (DLS) method is used to study it.

The measurements were performed on a setup assembled according to the traditional scheme [25]. Radiation from a 15 mW Ne-Ne laser was focused by a lens with a focus of 30 cm into a sample cuvette centered on the axis of the goniometer. A cylindrical cuvette, 15 mm in diameter with walls 0.8 mm thick, was placed coaxially in an immersion cylindrical quartz cuvette, 34 mm in diameter with walls 1.5 mm thick, filled with bi-distilled water to improve alignment. On the alidade of the goniometer, there was a photodetector based on an avalanche photodiode made by Photocor Ltd., Moscow, Russia. The signal from the photodetector was transmitted to a correlator from the same company [26], which constructed the intensity correlation function. The correlation functions were taken in the range of scattering angles *θ* from 35 to 120 degrees. At each angle, 3–4 functions were used.

### 2.4. Raman Spectroscopy

Raman spectra were measured in gels (2% by mass in water) and in air-dried samples of gels of alginate, pectin, and their mixture in the proportion 1:1 on quartz substrates. The measurements in gels were performed using an RS spectrometer In Via Raman Microscope (Renishaw, Gloucester, UK) [27] equipped with a diode laser with a wavelength of 785 nm and power at the cuvette inlet of 45 mW. RS spectra were recorded in a backscattering scheme. A 27 mm diameter cuvette was filled with an 8–10 mm-thick gel layer, and the cuvette was placed on the RS spectrometer microscope stage. The laser beam wasdirected vertically downward and was focused inside (deep into) the cuvette through the free surface of the gel using an NPlan 50/0.50 lens with a focal length of 8 mm (Leica, Wetzlar, Germany). The recording time was 200 s. For each sample, the spectrum was scanned 10 times, and the length of each scan was 20 s. The spectra measured in each experiment were processed and analyzed using Renishaw software WiRE 5.5 (RenishawFixture-Builderx64) and Origin 8.5. Measurements of dried gel samples on substrates were performed in the same scheme, but the laser beam was focused on the dried gel layer.

### 2.5. Cell Viability

For the in vitro study, solutions of sodium alginate 20 mg/mL, pectin 20 mg/mL, and alginate/pectin mixture at a concentration of 10 + 10 mg/mL were prepared. For this purpose, the corresponding polysaccharide suspensions were sterilized by autoclaving and then dissolved in DMEM/F12 medium (1:1), supplemented with 100 U/mL penicillin/streptomycin and 2 mM L glutamine.

Postnatal human dental pulp stem cells (DPCS) were isolated from the rudiment of a third molar extracted for orthodontic indications, as described previously [28], after the patient’s parents signed an informed consent form. All experiments were performed following the principles of Good Clinical Practice (GCP) and the ethical principles outlined in the current version of the Declaration of Helsinki. Cells were grown in DMEM/F12 medium (PanEco, Moscow, Russia), and supplemented with 10% fetal calf serum FBS (HyClone, Logan, UT, USA) in a humidified incubator at 37 °C and 5% CO_2_. The medium was changed after 24 h in the primary cell culture. Cells were incubated until dense growth islands or cell monolayer formation and then passaged for growth. Cells from the fourth passage were used for this study.

Cell viability in the presence of gels was analyzed using the direct contact method. For this purpose, cells were seeded in the wells of a 24-well plate at a concentration of 25 thousand cells/cm^2^ in DMEM/F12 (PanEco, Moscow, Russia) medium (1:1) with the addition of 10% fetal calf serum (FBS) (HyClone, Logan, UT, USA) and 100 U/mL penicillin/streptomycin and 2 mM L-glutamine), and were cultured at 37 °C in an atmosphere of 5% CO_2_. After 24 h, the medium was replaced with gels of pectin, sodium alginate and their mixture, which were placed in the cells of a 24-well plate and cultured at 37 °C in an atmosphere of 5% CO_2_ for 24 h.

The number of attached DPSCs was assessed after 1 day through differential fluorescence staining of live and dead cells using fluorescent dyes SYTO 9 (absorbance 420 nm, emission 580 nm) and propidium iodide (absorbance 488 nm, emission 640 nm). Microphotography was conducted on an Axiovert 200 (Zeiss, Jena, Germany) inverted microscope. The fluorescent dye SYTO 9, with an excitation wavelength of λ_excit_ = 450–490 nm and emission wavelength of λ_emis_ = 515–565 nm, stained the DNA and RNA of living and dead cells green. The intercalating reagent propidium iodide, with an excitation wavelength of λ_excit_ = 546 nm and emission wavelength of λ_emis_ = 575–640 nm, stained the nuclei of dead cells red.

The counting of viable cells was performed in a fixed surface area (micrographs) by determining the number of cells stained with SYTO 9 (representing all cells) and PI (indicating dead cells).

## 3. Results and Discussion

Alginate and pectin are two common polyuronates. Alginate is typically derived from seaweed, while pectin is obtained from citrus peel or apple pith. Alginate is a linear copolymer of (1 → 4)-β-linked-D-mannuronic acid (M) and α-L-guluronic acid (G), featuring a block distribution pattern with M-, G-, and MG-blocks [29]. On the other hand, the structure of pectin is considerably more complex. It is generally accepted that homogalacturonan (HG) and rhamnogalacturonan I (RG-I) are the primary building blocks of pectin [30]. In some pectins, Rhamnogalacturonan II (RG-II) is also present. HG, known as the “smooth” region of pectin, is a linear polymer made of (1 → 4)-α-linked D-galacturonic acid residues that are partially esterified with methyl by C-6 [27]. RG-I and RG-II, referred to as the “hairy” regions of pectin, have a main chain of (1 → 4)-α-linked D-galacturonic acid interrupted by (1 → 2)-α-linked L-rhamnopyranosyl residues. They also carry several branched side chains predominantly composed of neutral sugars, such as arabinose and galactose [31,32].

### 3.1. AFM Results

**Figure 1:**
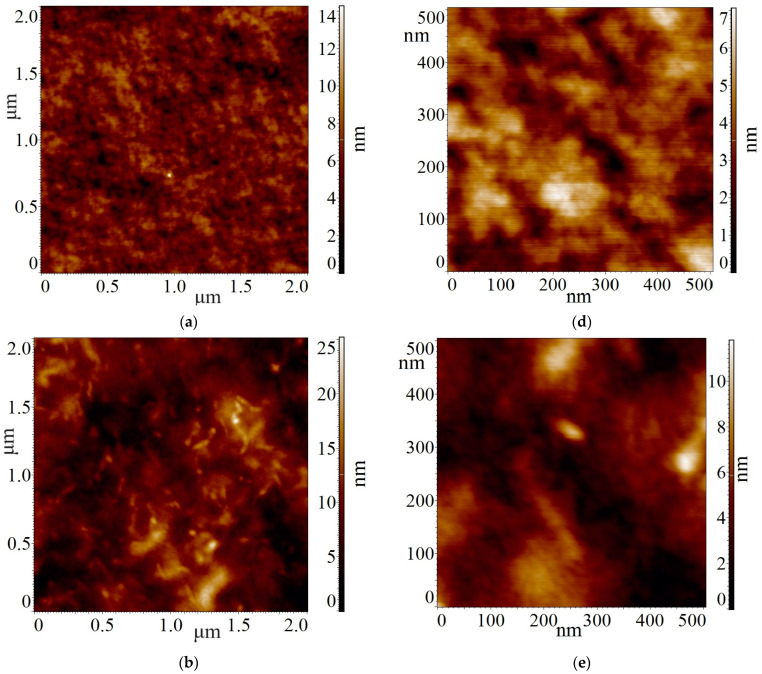

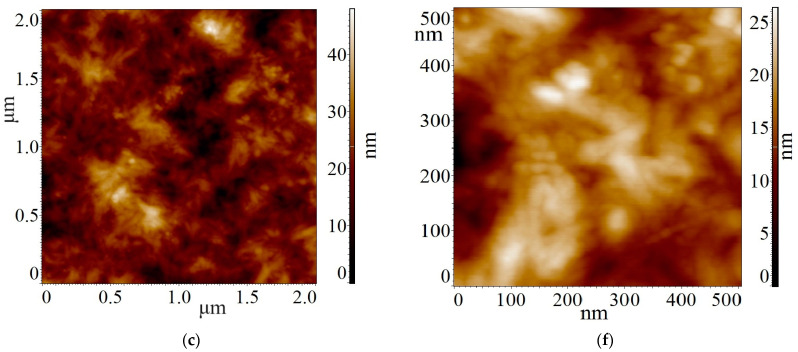
AFM images of dried gel samples on mica substrate. (**a**–**c**): scale 2 × 2 μm^2^; (**d**–**f**): 500 × 500 nm^2^; (**a**,**d**): alginate; (**b**,**e**): pectin; (**c**,**f**): 1:1 mixture.

During the experiment, the surface topography (relief) of four samples was examined by atomic force microscopy in intermittent-contact mode. The samples included a substrate (isinglass-stone), a film of vacuum-dried alginate gel (A), a film of pectin gel (P), and a dried gel film of mixed composition (alginate + pectin, 1:1) (AP). AFM images of the surface with dimensions of 5 × 5 μm^2^, 2 × 2 μm^2^, and 200 × 200 nm^2^ were obtained. The most representative images are those of 2 × 2 μm^2^ and 500 × 500 nm^2^, as shown in Figure 1.

It should be noted that the rounding radius of the cantilever tip used in the experiment does not exceed 10 nm. Therefore, it is possible that the actual lateral dimensions of the nano-grains may be smaller. Since the surface of the chipped mica used as a substrate is atomically smooth (*R_a_* does not exceed 0.2 nm), its contribution to the topography of the deposited films can be neglected.

An examination of the surface of samples of the alginate (A, Figure 1a,d), pectin (P, Figure 1b,e) and their mixture (AP, Figure 1c,f) revealed a significant increase in surface roughness on the micro scale compared to the substrate topography roughness. The substrate surface roughness (*R_a_*) was measured to be 0.21 nm for the image of 2 × 2 μm^2^. In contrast, for samples A, P, AP, the roughness values were 1.06, 2.63, and 4.67 nm, respectively. Thus, the topography roughness of A and P differ by a factor of 2.5, whereas in the mixture, it increased by a factor of 4.5 and 1.8, respectively.

A detailed comparison of the surface nano-relief of these three films is particularly interesting. An analysis of images (2 × 2 μm^2^ and 500 × 500 nm^2^) of the surface of these three films revealed a substantial difference in surface morphology among A, P, and their mixture.

Thus, the surface of the alginate film is predominantly flat, featuring pits of rounded shape with lateral dimensions and depth not exceeding 100 nm and 5 nm, respectively. The surface is characterized by a densely packed granular structure consisting of rounded grains with a diameter of approximately 10–20 nm (potentially smaller, considering that the size of the cantilever tip may overlap at these dimensions) (Figure 1a,d).

The pectin film exhibits two types of surface morphology. Images of 2 × 2 μm^2^ reveal the presence of anisotropic particles, measuring 40–50 nm in width and up to 200 nm in length. The height of these particles does not exceed 10 nm, and their density is approximately 20 per 1 × 1 μm^2^. Further analysis of images at 500 × 500 nm^2^ and 200 × 200 nm^2^ indicates that, similar to alginate, the film comprises densely packed particles, predominantly rounded and with a diameter of about 10–20 nm. Some of these particles may show slight anisotropy, with a width of about 10 nm and a length of approximately 20 nm (Figure 1b,e).

The film with a mixed composition (alginate + pectin) displays significant differences in surface topography. This sample features a fibrous (filamentous) surface structure, observable in both micron and submicron scale images (Figure 1c,f). Image analysis reveals that the fibers are randomly oriented, tightly packed, 10–50 nm wide, with a length independent of width ranging from 100–250 nm. These fibers exhibit a tendency to form structures at the 400-nanometer scale. Additionally, there are a small number of rounded particles with diameters of 10–50 nm. The mixed films demonstrate a quasi-square structure resembling that observed in [22] for pectin, which was not evident at the concentrations used in our pectin samples.

### 3.2. DLS Results

The relaxation of fluctuations in the fine structure of gels was investigated with the DLS method using the form of correlation functions of the intensity of light scattered by these gels.

**Figure 2:**
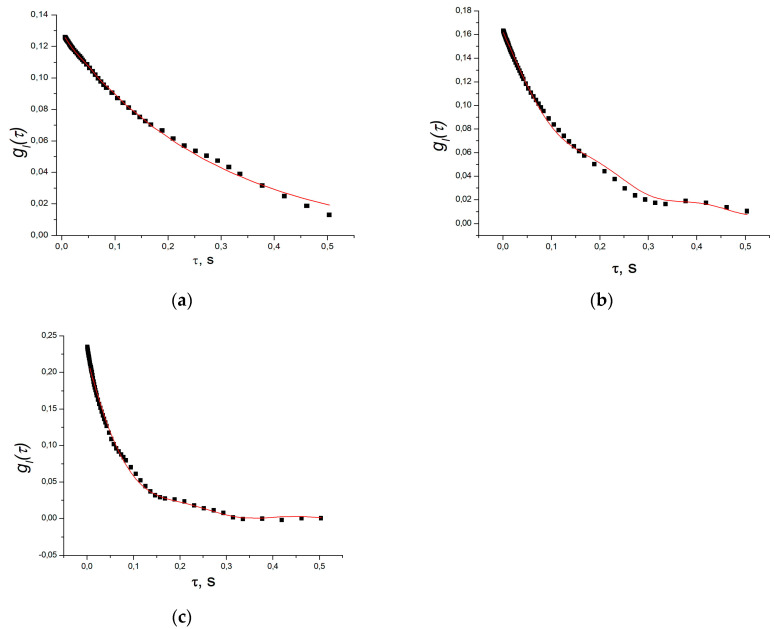
Correlation functions of light intensity scattered in pectin hydrogel (concentration 2% by mass) at angles: (**a**) at 45°, the curve is result of fitting by exponent to clearly show the presence of cosine component; (**b**,**c**) at 60° and 120°, respectively, curves are result of fitting by the Formula (1).

Figure 2 shows examples of correlation functions obtained in pectin at scattering angles of 45°, 60°, and 120°. The red curves represent attempts at fitting using a simplified formula:

(1) $$g_{I}\left(\tau \right)-1=Aexp(-2\Gamma \tau )+Bexp(-\Gamma_{2}\tau )cos\left(\omega_{s}\tau \right),$$

or, as specified in the figure caption, in some cases, an exponent was included in the fitting to highlight the presence of a cosine wave. This fitting approach aimed to estimate the average relaxation time of fluctuations in the gel structure $\tau_{g}=1/\Gamma$ and the frequency of elastic fluctuations $\omega_{s}$.

**Figure 3:**
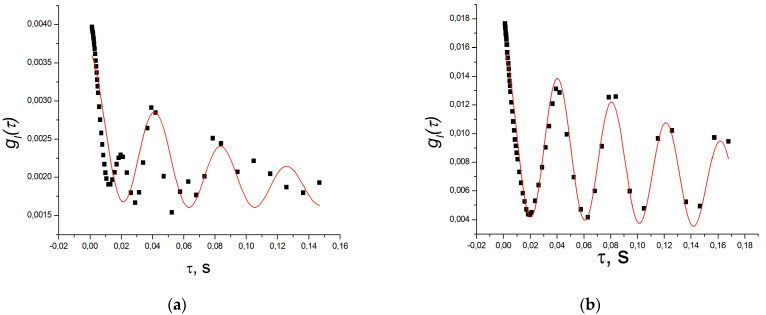
Correlation functions of light intensity scattered in alginate hydrogel (concentration 2% by mass) at angles: (**a**) at 45°; (**b**) at 120°, respectively, curves are result of fitting by the Formula (1).

Full convergence of the fitting process is not attainable due to the incomplete adequacy of the formula and a high number of parameters. However, from the figures and the tables (Table 1 and Table 2) below, it is evident that the average relaxation time typically decreases with an increasing scattering angle, in accordance with [33]. The frequency shift of the acoustic mode in the pectin hydrogel remains constant, exhibiting no dependence on the angle, with a value of $\omega_{s}$ = 27.1 ± 3.0 s^−1^ (period T = 0.23 ± 0.03 s). This angle-independent shift is even more pronounced in the example of scattering in an alginate gel (Figure 3 and Table 1). For alginate, the period of the “main” cosine is significantly smaller, measuring T = 0.040 ± 0.002 s, corresponding to $\omega_{s}$ = 158.3 ± 4.1 s^−1^.

Furthermore, at small scattering angles, an additional oscillation with a shorter period of about 0.02 s becomes apparent in the function. This further complicates the description of the correlation function.

**Figure 4:**
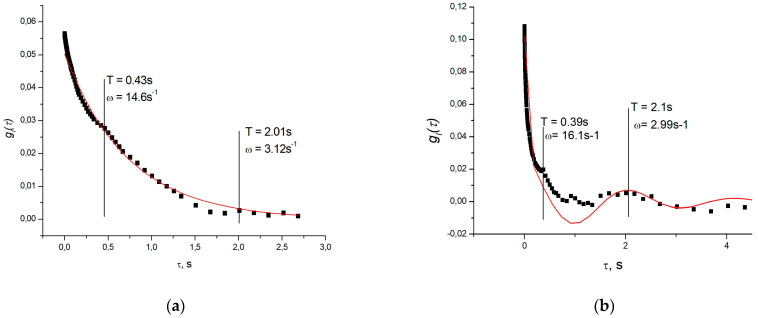
Correlation functions of light intensity scattered in the hydrogel of a 1:1 mixture of alginate and pectin (mixture concentration 2% by mass) at angles: (**a**) at 45°, the curve is fit by exponent for clarity of the cosine component presence; (**b**) at 120°, fit using Formula (1).

However, the primary focus of this work is on the alterations in the properties of the alginate–pectin mixture in comparison to its individual components. Examples of correlation functions of light scattered by the mixture are presented in Figure 4, and a comprehensive summary of the results is provided in Table 2.

Usually, the DLS method consists of determining the broadening of the central (unbiased) line of the spectrum of light scattered by a suspension of nanoparticles and determining the particle sizes based on this broadening. In this case, a correlator is used to determine the temporal correlation functions of the intensity and field of the scattered light. The latter is related to the scattered light spectrum by Fourier transform [34].

When light is scattered by monodisperse particles, the spectrum has a Lorentzian shape with a half-width Γ, and the temporal correlation function of the scattered light field *E* reflects the relaxation of the concentration fluctuations in non-interacting particles and is an exponent with a decay rate Γ, [35]:

(2) $$g_{E}\left(\tau \right)=\langle E\left(t\right)E^{*}\left(t+\tau \right)\rangle /\langle I\rangle =exp\left(-\Gamma \tau \right),$$

where *I* is the average intensity of light, *E* is the complex electric field of light, $g_{E}\left(\tau \right)$—is the correlation function of the field; the angle brackets denote time averaging, and the asterisk denotes complex conjugation. This form is due to the Brownian motion of particles.

The correlator constructs an intensity correlation function related to the field correlation function by the Siegert relation:

(3) $$G_{I}\left(\tau \right)=I^{2}\left(1+A{\left|g_{E}\left(\tau \right)\right|}^{2}\right)=I^{2}(1+Aexp(-2\Gamma \tau ))$$

Here *A* < 1 is a coefficient determined by the parameters of the optical system.

The correlator is used to determine the coherence time of the intensity correlation function *τ_c_* = 1/Γ and, from it, the average particle radius *r_p_* is:

(4) $$\Gamma =Dq^{2}=\frac{k_{B}Tq^{2}}{6\pi \eta r_{p}};\;q=\left|k_{s}{-k}_{L}\right|=\frac{4\pi n\sin\frac{\theta }{2}}{\lambda }$$

Here *q* is the scattering vector, $k_{s}{,\;k}_{L}$ are wave vectors of scattered and incident light, n is refractive index of medium, *λ* is wavelength of incident light, *θ* is scattering angle, $k_{B}$ is Boltzmann constant, *T* is absolute temperature, *η* is shear viscosity, *D* is particle diffusion coefficient.

In scenarios where a suspension or emulsion comprises particles of various sizes, the time correlation function becomes the sum of several exponentials. Consequently, the spectrum in such cases is the sum of Lorentzians. In contemporary pharmaceuticals, the acquired function is analyzed using a program for exponent decomposition with Tikhonov regularization. Additionally, a histogram of the scattering intensity distribution by particle sizes (specifically, by coherence times *τ_ci_* = 1/Γ*_i_*) is generated by the program, immediately recalculating these values into particle radii using Formula (5).

In the case of light scattering in gels, the situation is considerably more complicated. During the gel formation process, the diffusion exponent in the correlation function becomes blurred [36]:

(5) $$g_{I}\left(\tau \right)=G_{I}\left(\tau \right)/I^{2}=1+{A\left[Cexp\left(-2\Gamma \tau \right)+\left(1-C\right)exp\left[-{\left(\tau /\tau_{s}\right)}^{\beta }\right]\right]}^{2}$$

Here, *C* is the residual fraction of the contribution of diffusing particles; the appearance of the degree *β* corresponds to the appearance of the distribution of relaxation times with a form close to Gaussian; $\tau_{s}$ is the mean time of this distribution.

In the formed gel, the correlation function is expected to mirror its self-similar fractal structure, exhibiting both exponential and power forms [36,37]:

(6) $$g_{I}\left(\tau \right)=G_{I}\left(\tau \right)/I^{2}=1+{A\left(Cexp\left(-2\Gamma \tau \right)+\left(1-C\right)(1+\tau /\tau^{\prime }\right)}^{-2\varphi })$$

The determination of the fractal dimension is beyond the scope of this work, although the relationship between the degree φ and the fractal dimension of the gel was established in [38].

It is important to note that attempting to decompose the power correlation function into exponentials using any commercial programs attached to DLS devices will result in a size distribution depicted as a series of peaks uniformly located on the logarithmic scale of relaxation times. However, this distribution does not correspond to reality, and the particles with the obtained sizes are not necessarily present in the gel.

The theories mentioned above only describe the relaxation part of the correlation function, whereas gels exhibit significant elasticity, and both longitudinal and shear waves must propagate within them. In local media like liquids where all processes at some point depend on medium parameters only at this point, all possible waves are excited in a fluctuating manner, and fluctuations with different wavelengths are observed when the scattering angle is changed (Mandelstam–Brillouin scattering). The frequency shift in the scattered light is determined by:

(7) Δω=qV

where *V* is sound speed.

The gel is a nonlocal medium with strong coupling between neighboring regions. Consequently, stable modes of acoustic oscillations appear in the gel vessel, influenced by both the elastic properties of the gel and the characteristics of the vessel [39]. If the characteristic periods *T* (inverse frequencies) of such modes are significantly larger than the characteristic recession times of the correlation function (5) or (6), they manifest as small cosine oscillations at the tail of the correlation function (refer to, for instance, Figure 5 in [40]). In our case, using aqueous polysaccharide gels with concentrations of 2%, these times unfortunately coincide closely, significantly complicating the analysis of the functions. When pronounced cosinusoids corresponding to two acoustic modes are present, fitting programs simply refuse to minimize the fitting error in the presence of seven or nine fitting parameters. Therefore, the relaxation time was determined from the 1/e level of the correlation function, and each cosinusoid was fitted separately. RMS deviations, determined from 3 to 4 measurements, are provided as errors.

Table 2 indicates that the average relaxation time of the gels corresponds to the relation

(8) $$\tau =1/\Gamma ={\left(D_{g}q^{2}\right)}^{-1},$$

where, $D_{g}$ is no longer the diffusion coefficient, but some combination of elastic moduli [29]. These moduli of the mixture are close to the elastic moduli of the pectin hydrogel. Note that the deviation from this law in alginate at large scattering angles is due to a strong increase in the cosine component of the correlation function. This growth leads to the fact that, instead of Γ, approximation gives a value close to ${\left(T/4\right)}^{-1}$. 

Additionally, it can be observed that all our gels exhibit several stable acoustic modes, but in the mixture the “main” cosine with a period ranging from 1.45 to 2.1 s becomes prominent. It significantly exceeds the values of the periods of the cosine components, although the second, less pronounced cosine has a period of about 0.4 s, which is noticeably longer than in pectin, but close to the period of the main cosine in alginate. These periods correspond to $\omega_{s}$ = 2*π*/*T* = 3.1 ÷ 4.4 s^−1^ and $\omega_{s}$ = 13.2 ± 1.5 s^−1^. This may indicate either a significant decrease in the elastic moduli of the mixture, which contradicts the data on relaxation times, or a reduction in the attenuation in the mixture and the inclusion of slower acoustic modes due to the decrease in the damping of oscillations. Moreover, at an angle of 45°, a mode with a period of about 5 s is observed ($\omega_{s}$ = 13.2 ± 1.5 s^−1^, marked with an asterisk in the table). All of this may be attributed to the higher cohesion of the gel formed by the mixture compared to the component gels. This is also indicated by the appearance of “threads” on AFM images of the mixture.

We can attempt to elucidate the nature of this increase by investigating the change in the Raman spectra.

### 3.3. RS Results

**Figure 5:**
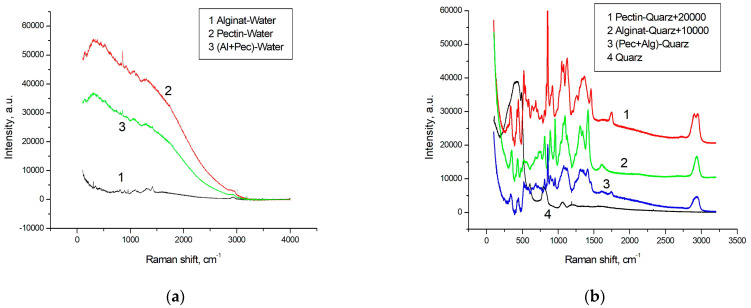
(**a**): RS spectra of hydrogels of alginate, pectin and their 1:1 mixture after subtracting the RS spectrum of water, concentration of gel-forming agent in water 2% by mass; (**b**): spectra of air-dried samples of the same hydrogels on a quartz substrate after subtracting the RS spectrum of the quartz substrate and the RS spectrum of the quartz substrate.

Raman spectra obtained for gels (after subtracting the RS spectrum of water) and for dried gel samples (after subtracting the RS spectrum of quartz substrate) are shown in Figure 5a,b, respectively.

**Figure 6:**
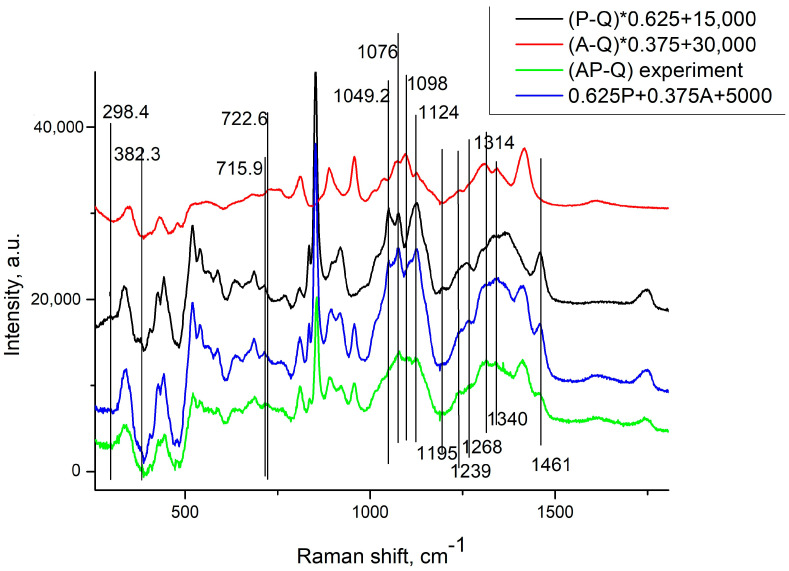
RS spectra of pectin and alginate (after subtraction of the substrate Q spectrum) with coefficients of 0.625 and 0.375, respectively, their sum, and the RS spectrum experimentally obtained in the mixture. P means pectin; A means alginate; AP means mixture of pectin and alginate; Q means quartz.

In Figure 5, it can be seen that the Raman spectrum of pectin and, consequently, of the mixture, is situated on the luminescence mount, whose spectrum is well described by two Gaussians, similar to the approach used to describe the luminescence of milk fats and proteins in [41,42]:

(9) $$I_{Lum}=A_{1}exp(-2((x-x_{c1})/w_{1}{)}^{2})+A_{2}exp(-2((x-x_{c2})/w_{2}{)}^{2}),$$

where $A_{1}$ = 40,981, $A_{2}$ = 36,967, $w_{1}$ = 881.9 cm^−1^, $w_{2}$ = 1323 cm^−1^, $x_{c1}$ = 235.4 cm^−1^, $x_{c2}$ = 1323.1 cm^−1^. The background luminescence of the mixture is 1.6 times weaker. However, alginate hardly luminesces at all. After subtracting the luminescence, these spectra appear to be highly noisy. Hence, we utilized the Raman spectra of dried samples for subsequent analysis, following the approach in [43,44,45]. It is important to note that the final objective of the entire project is the development of sponges for therapeutic patches. Consequently, alterations in the dry samples were of greater interest to us than those in the wet ones. Furthermore, nearly all the Raman lines of interest exhibited similar behavior in both aqueous and dried samples during the mixing of components. The ratio of luminescence intensities of 1.6:1 in the pectin hydrogel and in the hydrogel of the mixture, in the absence of luminescence in alginate, gives us a ratio of molecular concentrations of pectin and alginate in the mixture of 0.625/0.375. Figure 6 shows the measured spectra of pectin and alginate (after subtraction of the substrate spectrum) with the indicated ratios, their sum, and the experimentally obtained RS spectrum in the mixture for ease of comparison. Note that the spectra obtained for both alginate and pectin practically coincide with the data in the literature [43,44,45,46].

In the spectra of sodium alginate (A), the band at 2939 cm^−1^ refers to CH-bond vibration. The peak band at 1613 and 1418 cm^−1^ corresponds to the asymmetric and symmetric stretching band in the carboxyl (-COOH) group [47,48]. Three bands are present in the 1400–1270 cm^–1^ region. The band at 1340 cm^–1^ refers to the deformation vibration of C-H in MG, the band at 1309 cm^–1^ refers to the valence vibration of C-O, and the one at 1239 cm^−1^ refers to stretching in the glycosidic C-O-C bond [11,32,47,48]. Alginate spectra show several bands in the 1200–950 cm^–1^ region; this region is associated with C-OH deformation, C-C-H bending, C-O and C-C valence vibrations, and each band may be attributed to the contribution of two or more types of motions [44,49,50]. The band at 1098 cm^–1^ is attributed to mannuron links. The region between 950 and 750 cm^−1^ is referred to as the “fingerprint” or anomeric region. In the spectra of alginate samples, a band at 812 cm^−1^ is related to skeletal stretching and deformation modes, and two more bands at 725 and 759 cm^−1^ are associated with ring respiration in the mannuron–guluron and mannuron–mannuron chain fragments [50]. The bands at 679, 483, and 430 cm^−1^ pertain to the deformation of pyranosyl rings and C-O-C glycosidic bond vibrations.

Pectin is a linear polysaccharide containing D-galacturonic acid as the major component; the units of D-galacturonic acid are connected by glycosidic bonds. In the spectrum of pectin, the intense band at 2952 cm^−1^ is attributed to the stretching of the C-H bond in the pyranose ring carbon atoms, while the bands at 2901 and 1458 cm^−1^ can be identified as symmetric and asymmetric internal vibrations in the methyl group in esters. The observed bands at 1747 and 1371 cm^–1^ result from vibrations of esterified pectin groups [51]. The peaks at 1371 and 1336 cm^−1^ correspond to the bands of deformation vibrations in CH, and the peak at 1268 cm^−1^ corresponds to the valence vibrations in the C=O bond in the acetyl group. Peaks at 1125, 1079, and 1049 cm^−1^ correspond to valence vibrations in C-O bonds (-C-O- stretch of carbohydrates), while peaks at 918 cm^−1^ pertain to pendular, complex ester vibrations in CH_3_. The clear band at 854 cm^−1^ can be considered as a marker band for α-glycosidic bonds in pectin [44]. The bands at 760, 682, 635, and 517 cm^−1^ correspond to vibrational modes, while those at 726, 440, and 424 cm^−1^ correspond to deformation vibrations in the pyranose ring.

Homogeneous mixed gels are obtained using compatible polymers, i.e., polymers capable of binding. Polysaccharide associations usually produce synergistic effects that can be observed through the properties of the systems (gel strength, viscosity, etc.). However, synergistic interactions with alginate are relatively rare, even though, from a molecular perspective, alginate is capable of forming hydrogen bonds with various polysaccharides [52]. So far, synergism has been identified with pectin and xanthan gum [53].

When comparing the experimentally obtained alginate/pectin spectrum with the sum of the measured spectra of alginate and pectin with the indicated ratios, it is evident that the RS spectrum of the mixture resembles the sum of the spectra of pectin and alginate [54].

However, the bands related to vibrational (517, 635 cm^−1^), deformation (716 cm^−1^), and valence vibrations (1049 cm^−1^) in the pectin pyranose ring bonds, as well as the bands at 725 and 759 cm^−1^ caused by ring respiration in mannuron–guluron and mannuron–mannuron chain fragments, and the band at 1095 cm^−1^ caused by mannuron links, practically disappeared in the spectrum of the mixture. The spectral band of pectin at 716 cm^−1^, corresponding to C-O-C deformation vibrations in the pyranose ring, is slightly shifted to the right, and the band at 1049 cm^−1^, representing the valence vibrations in the pyranose ring of pectin, practically disappears. The spectral band at 1268 cm^−1^, corresponding to valence vibrations in the C=O bond in the acetyl group, disappears. The 1124 cm^−1^ band, corresponding to the C-OH stretch in both alginate and pectin, is strongly attenuated. The weak line of alginate at 1239 cm^−1^, representing stretching in the glycosidic C-O-C bond, becomes more pronounced. However, the alginate’s 1340 cm^−1^ peak, corresponding to the C-H strain fluctuations in MG, is strongly reduced.

**Figure 7:**
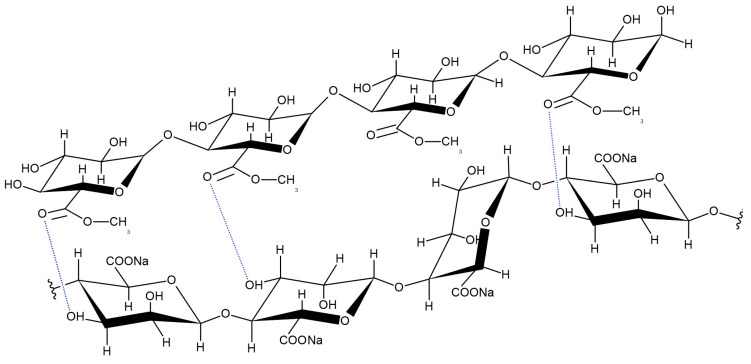
Schematic illustration of chemical structures of pectin and alginate based on Raman spectra, created using the ACD/ChemSketch program (version 14.00) Hydrogen bonding are denoted by blue dotted lines.

Thus, it can be considered that the two biopolymers in the mixture are linked together by hydrogen bonds between the hydroxy group of the mannuronic link of sodium alginate and the carboxyl group of pectin (see Figure 7), without altering their chemical structure [54].

### 3.4. Biocompatibility Tests

**Figure 8:**
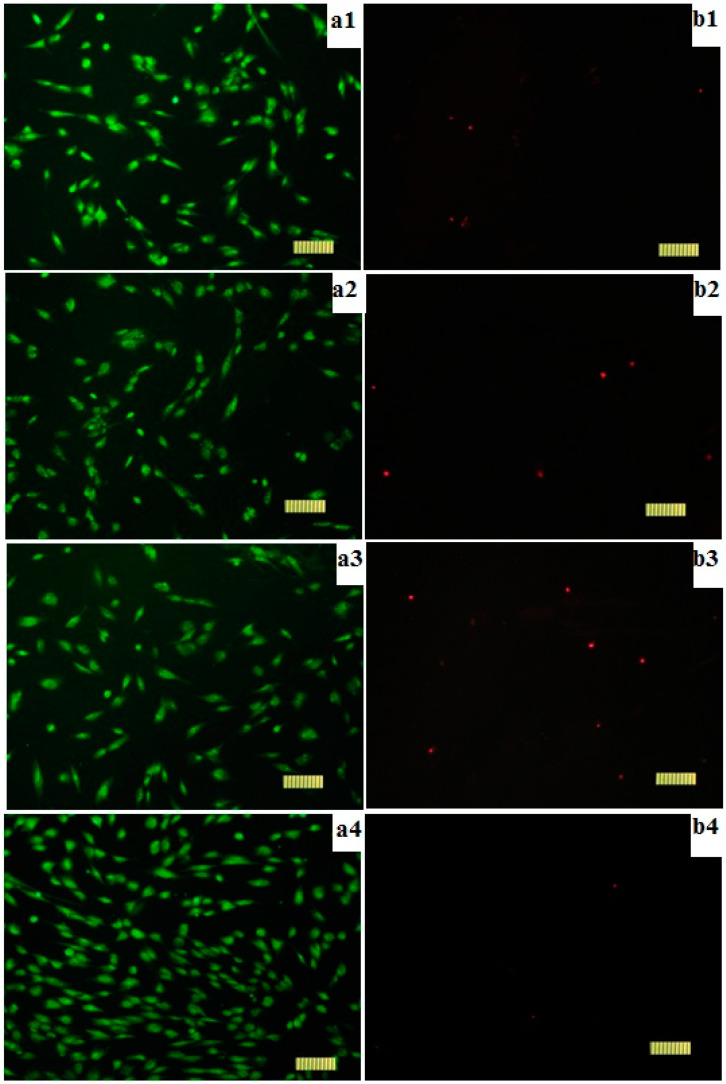
Appearance of cells cultured in the presence of gels. SYTO 9 (**a**) and PI (**b**) staining, 100 μm scale. 1—alginate, 2—pectin, 3—alginate/pectin, 4—control.

Testing the biocompatibility of medical devices and drugs is crucial to ensure their safe use, especially in humans. In vitro cytotoxicity can indicate changes within cells, ranging from cell death to minor alterations in some cellular activities. Evaluation of the viability of the DPSC cells cultured in the presence of gels, conducted using the method of differential fluorescence staining of live and dead cells, allowed us to establish the absence of significant differences between the effects of the investigated gels. Characteristic images of cells cultured in the presence of the studied gels are presented in Figure 8.

The cell viability was more than 98% (Figure 8, Table 3), indicating that alginate, pectin, and their mixture are biocompatible.

The lack of cytotoxicity of alginate/pectin gels is in agreement with the results obtained in [10] for the immortalized human keratinocyte (HaCaT) cell line.

## 4. Conclusions

The objective of our study was to explore the potential use of a hydrogel mixture of alginate and pectin in water for the development of novel wound healing formulations. Additionally, we aimed to understand the alterations in the properties of such a gel in comparison to gels formed by its individual components. The interaction between sodium alginate and pectin within the gel of their mixture was investigated, and the biocompatibility of such a gel was demonstrated. Our findings revealed that the gel structure undergoes significant changes in a dried mixture of alginate and pectin with a 1:1 ratio, presenting a highly complex structure compared to the gels formed of individual components (Figure 1). A fibrous (filamentous) structure emerges, observable at both the micron and submicron scales. These fibres exhibit a random orientation, dense packing, a width of 10–50 nm, a consistent length of 100–250 nm, and a tendency to form 400-nanometer-scale structures.

Furthermore, the disturbance wave attenuation in the mixture gel was shown to decrease compared to the gels made from individual components, incorporating slower acoustic modes due to reduced oscillation attenuation. Notably, acoustic modes with periods of several seconds become apparent in the mixture gel (Figure 2, Figure 3 and Figure 4 and Table 2).

Raman spectroscopy provided insights into the changes occurring in the molecular structure of the studied materials. The analysis revealed the appearance of additional HO-O bonds in the gel of the mixture (Figure 7), leading to modifications in the structure, dynamics, and viscoelastic properties of the mixture gel (Figure 2, Figure 3 and Figure 4 and Table 2). Despite these alterations, the biocompatibility of the mixture gel is comparable to that of gels formed by individual components.

The viability of DPSC of approximately 98% indicated that alginate, pectin, and their mixture gels are biocompatible.

## Figures and Tables

**Table 1:** Frequencies of gel vibrations $\omega_{s}$ observed at different scattering angles *θ* in inverse seconds.

*θ*	Alginate (A)	Pectin (P)	Alginate + Pectin (AP)
35°	162.5 ± 2	31.7 ± 7.7	-
	311 ± 11	-	-
45°	153.5 ± 6.0	25.7 ± 5.5	3.36 ± 1.35
	309 ± 9	-	11.9 ± 1.3
60°	162.2 ± 1.2	31.5 ± 1.4	4.4 ± 0.4
	321 ± 14	-	11.4 ± 2.0
120°	155.0 ± 0.9	24.0 ± 3.2	3.12 ± 0.16
	-	-	12.1 ± 1.6

**Table 2:** Parameters of hydrogels of alginate, pectin, and their 1:1 mixture. Concentration of gel-forming agent in water was 2% by weight.

Angle	Value	Alginate	Pectin	Mixture 1:1
45°	Γ (s^−1^)	82.6 ± 10.2	3.30 ± 0.25	3.64 ± 1.54
	Γ/q^2^ × 10^−9^ (cm^2^/s)	8.05	0.322	0.355
	$\omega_{s1}$ (s^−1^)	153.5 ± 6.0	25.7 ± 5.5	3.36 ± 1.35
	$\omega_{s2}$ (s^−1^)	309 ± 9	-	11.9 ± 1.3
	$\omega_{s3}$ (s^−1^)	-	-	1.26 *
	B/A	0.230 ± 0.008	0.033 ± 0.007	0.09 ± 0.02 0.491 *
60°	Γ (s^−1^)	146.3 ± 0.007	5.86 ± 0.17	5.46 ± 0.18
	Γ/q^2^ × 10^−9^ (cm^2^/s)	8.35	0.334	0.311
	$\omega_{s1}$ (s^−1^)	162.2 ± 1.2	31.5 ± 1.4	4.4 ± 0.4
	$\omega_{s2}$ (s^−1^)	321 ± 14	-	11.4 ± 2.0
	B/A	0.204 ± 0.032	0.033 ± 0.002	0.075 ± 0.016
120^°^	Γ (s^−1^)	118.7 ± 0.008	15.6 ± 0.06	17.4 ± 0.06
	Γ/q^2^ × 10^−9^ (cm^2^/s)	2.26	0.297	0.331
	$\omega_{s1}$ (s^−1^)	155 ± 0.9	24.0 ± 3.2	3.12 ± 0.16
	$\omega_{s2}$ (s^−1^)	-	-	12.1 ± 1.6
	B/A	0.340 ± 0.013	0.109 ± 0.03	0.103 ± 0.007

* The asterisk indicates the frequency of the ultra-slow mode and its relative amplitude.

**Table 3:** Results of cell viability studies of cells grown in the presence of gel.

Sample	Total Number of Cells per mm^2^	Number of Non-Viable Cells per mm^2^	Viability, %
Sodium alginate	198.6 ± 20.6	1.6 ± 1.3	99.2 ± 0.2
Pectin	187.4 ± 11.7	3.4 ± 1.7	98.2 ± 0.3
Sodium alginate/pectin	187.0 ± 9.7	3.4 ± 1.5	98.1 ± 0.2
Control	206.0 ± 16.4	0.4 ± 0.5	99.8 ± 0.1

## Data Availability

The data are available upon a reasonable official request to the corresponding authors.
